# Multi-regulatory potency of USP1 on inflammasome components promotes pyroptosis in thyroid follicular cells and contributes to the progression of Hashimoto's thyroiditis

**DOI:** 10.1186/s10020-024-00885-w

**Published:** 2024-08-12

**Authors:** Xuying Zhao, Wenyu Ni, Wenjie Zheng, Wenkai Ni, Chunfeng Sun, Yunjuan Gu, Zhifeng Gu

**Affiliations:** 1 Dalian Medical University, Dalian, Liaoning China; 2 Department of Endocrinology and Metabolism, Affiliated Hospital of Nantong University, No. 20 Xisi Road, Nantong, 226001 Jiangsu China; 3 Department of Endocrinology and Metabolism, Qidong People’s Hospital, Nantong, Jiangsu China; 4 Research Center of Clinical Medicine, Affiliated Hospital of Nantong University, Nantong, Jiangsu China; 5 Department of Gastroenterology, Affiliated Hospital of Nantong University, Nantong, Jiangsu China; 6 Department of Nuclear Medicine, Affiliated Hospital of Nantong University, No. 20 Xisi Road, Nantong, 226001 Jiangsu China; 7 Department of Rheumatology, Affiliated Hospital of Nantong University, No. 20 Xisi Road, Nantong, 226001 Jiangsu China

**Keywords:** USP1, Inflammasome, Pyroptosis, Thyroid follicular cells, Hashimoto's thyroiditis

## Abstract

**Background:**

Inflammatory diseases are often initiated by the activation of inflammasomes triggered by pathogen-associated molecular patterns (PAMPs) and endogenous damage-associated molecular patterns (DAMPs), which mediate pyroptosis. Although pyroptosis resulting from aberrant inflammasome triggering in thyroid follicular cells (TFCs) has been observed in Hashimoto's thyroiditis (HT) patients, the underlying mechanisms remain largely unknown. Given the extensive involvement of protein ubiquitination and deubiquitination in inflammatory diseases, we aimed to investigate how deubiquitinating enzymes regulate thyroid follicular cell pyroptosis and HT pathogenesis.

**Methods:**

Our study specifically investigated the role of Ubiquitin-specific peptidase 1 (USP1), a deubiquitinase (DUB), in regulating the inflammasome components NLRP3 and AIM2, which are crucial in pyroptosis. We conducted a series of experiments to elucidate the function of USP1 in promoting pyroptosis associated with inflammasomes and the progression of HT. These experiments involved techniques such as USP1 knockdown or inhibition, measurement of key pyroptosis indicators including caspase-1, caspase-1 p20, and GSDMD-N, and examination of the effects of USP1 abrogation on HT using a mouse model. Furthermore, we explored the impact of USP1 on NLRP3 transcription and its potential interaction with p65 nuclear transportation.

**Results:**

Our findings provide compelling evidence indicating that USP1 plays a pivotal role in promoting inflammasome-mediated pyroptosis and HT progression by stabilizing NLRP3 and AIM2 through deubiquitination. Furthermore, we discovered that USP1 modulates the transcription of NLRP3 by facilitating p65 nuclear transportation. Knockdown or inhibition of USP1 resulted in weakened cell pyroptosis, as evidenced by reduced levels of caspase-1 p20 and GSDMD-N, which could be restored upon AIM2 overexpression. Remarkably, USP1 abrogation significantly ameliorated HT in the mice model, likely to that treating mice with pyroptosis inhibitors VX-765 and disulfiram.

**Conclusions:**

Our study highlights a regulatory mechanism of USP1 on inflammasome activation and pyroptosis in TFCs during HT pathogenesis. These findings expand our understanding of HT and suggest that inhibiting USP1 may be a potential treatment strategy for managing HT.

**Supplementary Information:**

The online version contains supplementary material available at 10.1186/s10020-024-00885-w.

## Introduction

Hashimoto's thyroiditis (HT), also known as chronic lymphocytic thyroiditis or autoimmune thyroiditis, is characterized by lymphocyte infiltration and the presence of antibodies specific to thyroid antigens (Antonelli et al. 2015; Caturegli et al. 2014). HT is considered the leading cause of hypothyroidism, which can result in hyperlipidemia, arteriosclerosis, and cardiac hypertrophy (McDermott 2020; Chen et al. 2015). With a high morbidity rate, HT is one of the most common thyroid diseases (Ragusa et al. 2019); however, its etiology and pathogenesis are not fully understood. Genetic susceptibility, environmental triggers, and autoimmunity contribute to the occurrence and development of Hashimoto's thyroiditis (Ralli et al. 2020). Tissue-specific autoimmunity, particularly in the thyroid follicular cells (TFCs), is another significant contributing factor apart from immune cell homeostasis disequilibrium (Kawashima et al. 2011). Toll-like receptors expressed in TFCs respond to various pathogen-associated molecular patterns (PAMPs) and endogenous damage-associated molecular patterns (DAMPs), activating the innate immune system and recruiting self-reactive lymphocytes to the thyroid (Kawashima et al. 2011, 2013; Harii et al. 2005). Conversely, multiple pro-inflammatory cytokines produced by these lymphocytes, such as interferon-γ (IFN-γ), interleukin-17 (IL-17), interleukin-6 (IL-6), and tumor necrosis factor-α (TNF-α), cause injury to TFCs (Phenekos et al. 2004; Ganesh et al. 2011), resulting in the release of IL-1β, IL-18, and other cellular content, amplifying the inflammatory response and TFCs' injury (Ralli et al. 2020; Liu et al. 2010). Thus, the concomitant damage of TFCs along with the pro-inflammatory microenvironment may induce HT, and blocking this process could be a prospective strategy for HT management.

Pyroptosis is also known as inflammatory necrosis, a programmed cell death mediated by gasdermin and dependent on the activation of cytosolic inflammasomes (Shi et al. 2017). Inflammasomes are composed of NOD-like receptors (NLRs)/abscent in melanoma 2 (AIM2), apoptosis-associated speck-like protein containing a card (ASC), and the terminate effector pro-caspase-1 (Schroder and Tschopp 2010). Inflammasome activation involves the combination of inflammasome components and cleavage of procaspase-1 into an active subunit, which induces the maturation of interleukin-1β (IL-1β) and interleukin-18 (IL-18) and cleavage of Gasdermin D, subsequently initiating pyroptosis (Shi et al. 2017). Abnormal expression and dysfunction of inflammasomes are closely related to autoimmunity and organ damage (Shaw et al. 2011; Choulaki et al. 2015). Inflammasomes have been demonstrated mainly in peripheral monocytes, but recent studies have also identified their existence in tissue cells, such as islet-β cells, neurons, and keratinocytes (Oslowski et al. 2012; Kaushal et al. 2015; Dombrowski et al. 2011). Inflammasome activation in thyroid follicular cells of HT patients was firstly proposed in 2018, and more inflammasomes were observed in thyroid follicular cells at sites of inflammatory infiltration than that at areas without inflammatory infiltration (Guo et al. 2018). Excessive iodine promotes pyroptosis of TFCs in HT via the ROS-NF-κB-NLRP3 pathway, implying the relationship between inflammasome-dependent pyroptosis and HT development (Liu et al. 2019); however, the specific regulatory mechanisms remain largely unknown.

Inflammasome activation is modulated by various post-translational modifications, including deubiquitination, the reverse process of ubiquitination by removing ubiquitin from modified proteins (Man and Kanneganti 2015). Deubiquitinases (DUBs) are the most critical effectors driving the deubiquitination process, classified into seven distinct superfamilies: Ub C-terminal hydrolases (UCHs), Ub-specific proteases (USPs), ovarian tumor proteases (OTUs), Machado-Josephin domain proteases (MJDs), motif interacting with Ub-containing novel DUB family (MINDY), Jab1/Mov34/MPN + protease (JAMM) family, and zinc-finger-containing Ub peptidase (ZUP1) (Clague et al. 2019). Among them, USPs belong to the largest subfamily and exert the most complicated biological functions. This study aims to investigate potential USPs associated with inflammasome-related HT development. Mechanistically, we discovered the regulation whereby USP1 could interact and stabilize NLRP3 and AIM2, which further instigated inflammasomes and pyroptosis. Our findings demonstrate a novel role of USP1 in modulating pyroptosis and may provide a more comprehensive understanding of the pathogenesis of HT.

## Methods

### Animals

8-weeks-old female C57BL/6 mice were purchased from Laboratory Animal Center of Nantong University. The mice were maintained under standard conditions with controlled temperature, humidity, and a 12-h light/dark cycle. An emulsion was formed by dissolving 2 mg/ml bovine thyroglobulin in 0.01 M phosphate buffer saline, followed by mixture with an equal volume of complete Freund’s adjuvant (CFA) or incomplete Freund's adjuvant (IFA). To establish an HT mouse model, the mice were fed with 0.05% NaI water and accepted multiple injections of bovine thyroglobulin (200 μg/mouse) combined with CFA on the back, abdomen and neck in day 0 of modeling. After 2 weeks, they received regular injections of bovine thyroglobulin (200 μg/mouse) and IFA every 2 weeks. From the fourth week, the mice were divided into groups and received weekly intraperitoneal injections of ML323 (10 mg/kg), VX-765 (50 mg/kg), and disulfiram (50 mg/kg). The dosage of intraperitoneal injection was referred to the previous literature (Song et al. 2020; Jin et al. 2022; Hu et al. 2020). Each group consisted of six mice. After maintaining for 12 weeks, the mice were sacrificed, and their thyroids were collected for further analysis. All experimental procedures were approved by the Institutional Animal Care and Use Committee of Nantong University.

### Human thyroid samples

Thyroid samples were obtained from 24 female patients with benign thyroid nodules scheduled for thyroidectomy at the Affiliated Hospital of Nantong University. Thyroid specimens adjacent to the nodules were collected. The samples consisted of 12 normal thyroid tissues and 12 thyroid tissues from patients diagnosed with Hashimoto's thyroiditis (HT) based on postoperative pathology and positive serum thyroid peroxidase antibody. Out of the total, RNA and total protein were extracted from 7 pairs of tissues for subsequent analyses, while 5 pairs underwent sectioning post-paraffin embedding for histological examination. All research procedures were approved by Ethics Committee of Affiliated Hospital of Nantong University, and all participants provided their informed and written consent.

### Reagents

TNF-α and IFN-γ were purchased from R&D. Anti-NLRP3 (1:500 for WB, 1:100 for IHC), anti-NLRP1 (1:100 for IHC), anti-NLRC4 (1:100 for IHC), anti-TNF-α (1:100 for IHC) and anti-caspase-1 (1:500 for WB, 1:100 for IHC) were obtained from ABclonal Technology (Wuhan, China). Anti- AIM2 (1:100 for WB, 1:50 for IHC and IF), anti-ASC (1:50 for IHC), anti-IL-1β (1:50 for IHC) and anti-BAX (1:100 for WB) were from Santa Cruz Biotechnology (TX, USA). Anti-USP1 (1:1000 for WB, 1:100 for IHC), anti-IFN-γ (1:100 for IHC) and anti-GAPDH (1:1000 for WB) were from Proteintech. Anti-GSDMD-N (1:1000 for WB) was purchased from Abcam (Cambridge, UK). Anti- NF-κB (p65) (1:100 for IF, 1:1000 for WB), anti-caspase-3 (1:1000 for WB) and anti-cleaved caspase-3 (1:1000 for WB) was obtained from cell signaling technology (MA, USA). Protein A/G agarose was obtained from Bioepitope. Secondary antibodies for WB were purchased from Southern-Biotech (NJ, USA). Cycloheximide (CHX), complete Freund’s adjuvant and incomplete Freund’s adjuvant were from sigma (MO, USA). Bovine thyroglobulin was obtained from Bioss (MA, USA). ML323 was from Selleck Chemicals (TX, USA). VX-765 and disulfiram were purchased from MedChemExpress (NJ, USA). Product code and Research Resource Identifiers (RRIDs) were presented in Table S2.

### Cell culture

The Nthy-ori 3-1 cell line purchased from Zhong Qiao Xin Zhou Biotechnology (Shanghai, China) was cultivated in RPMI-1640 (Corning) supplemented with 10% fetal bovine serum (Gibco) and 1% Penicillin–Streptomycin (NCM Biotech) in the presence of 5% CO2 at 37℃. Cells were treated with recombinant human TNF-α at concentrations of 5 and 10 ng/ml or IFN-γ at concentrations of 12.5 and 25 ng/ml for 24 h.

### Plasmid and ShRNA preparation and transfection

Plasmids coding for USP1(p3XFLAG-CNV-10-USP1), enzyme activity mutated USP1(p3XFLAG-CNV-10-USP1 C90S) and AIM2 (pTSBX-CMV-AIM2 NM_004833.3-EF1-copGFP-2A-PURO), ShRNA targeting USP1 and control ShRNA were designed and purchased from TransheepBio-Tech CO, LTD (Shanghai, China). The authenticity of all the plasmids was verified by DNA sequencing. All transfections were conducted using Lipofectamine 3000 reagent (Invitrogen) according to the manufacturer’s instructions.

### Hematoxylin and eosin (H&E) staining and pathologic assessment

H&E staining were conducted to assess the pathological changes in human and mouse thyroid sections. Lymphocyte infiltration score was determined by 2 independent researchers according to the percentage of lymphocyte infiltration area as the followings: 0, normal; 1 + , 1–10%; 2 + , 10–30%; 3+, 30–50%; 4 + , > 50% (Li et al. 2017). Three fields of view were observed for each sample, and the average score was calculated.

### Immunohistochemistry, immunofluorescence

Paraffin sections of human and mouse thyroid tissues were processed for immunohistochemistry analysis. The sections were deparaffinized, rehydrated and incubated with sodium citrate buffer for antigen repair. After blocking by 5% bovine serum albumin for 1 h at room temperature, the sections were incubated with the indicated primary and secondary antibodies. Finally, the sections were counterstained with 3,3′-diaminobenzidine and hematoxylin and observed under an optical microscope. For cell immunofluorescence, the slides were fixed in 4% paraformaldehyde and 0.1% Triton X-100, then blocked with 5% bovine serum albumin for 30 min at room temperature. After incubation with the primary antibodies for 12 h, cell slides were washed and probed with Fluor–labeled secondary antibodies (ABclonal Technology) and DAPI (Cell signaling technology), following by visualization via confocal laser microscopy.

### RT-qPCR

Total RNA was extracted from cultured thyroid cells and human thyroid tissues using TRIzol reagent (Thermo). cDNA synthesis was performed using a qPCR RT Kit (Toyobo, Japan), and real-time PCR was conducted using the Fast SYBR Green Master Mix (Applied Biosystems Inc., MA) according to the manufacturer's instructions. The relative expression levels were determined using control values as references and analyzed statistically by GraphPad Prism version 7. The sequences of the primers were listed in Table S1.

### Immunoblotting, immunoprecipitation and protein stability assay

Thyroid tissues and cultured thyroid cells were lysed by RIPA lysis buffer (P0013B, Beyotime) with protease inhibitors (Roche) and phosphatase inhibitors (Roche). All samples were centrifuged and then boiled with SDS Loading Buffer (Beyotime). Protein molecules were separated by SDS gels and transferred to PVDF Membranes (Sigma). After blocking in 5% skimmed milk, the membranes were incubated with primary antibodies overnight at 4 °C, following by washing and probing with secondary antibodies (Southern-Biotech). The signals were detected using enhanced chemiluminescence (ECL) kit (Millipore). For immunoprecipitation, cells were lysed by RIPA lysis buffer (P0013C, Beyotime). Then the lysates were incubated with primary antibodies overnight at 4 °C, following by incubating with protein A/G agarose for 2.5 h at 4 °C. Subsequently, the proteins were eluted from the beads and subjected to immunoblotting. For protein stability assay, cells were administrated with CHX (100 μg/ml) and collected at different time points, thereby conducting western blotting to detect the protein degradation.

### LIVE/DEAD assay

The LIVE/DEAD Viability/Cytotoxicity kit (Thermo) were used as described by the manufacturer. Stained cells were immediately viewed under a fluorescent microscope.

### Enzyme-linked immunosorbent assay (ELISA)

A total of 1 ml of whole blood was collected using retrobulbar bleeding method under anesthesia in an EP tube. After standing for 2 h at 37 ℃, the whole blood was centrifuged at 4000 rpm for 10 min at 4 ℃ to obtain serum. The concentration of thyroglobulin antibodies (TGAb), thyroid peroxidase antibody (TPOAb) and thyroid stimulating hormone (TSH) in mouse serum was determined by ELISA using commercial quantitative kits (CUSABIO and Jianglaibio) according to the manufacturer’s recommended protocols.

### Bioinformatic analyses

Microarray data of GSE138198-GPL6224 (13 HT tissues and 3 normal tissues) was obtained from Gene Expression Omnibus (GEO) database (http://www.ncbi.nlm.nih.gov/geo/). The analysis of DEGs was performed using limma R package. DEGs with adjust p < 0.05 and log2 fold change (FC) (log2FC) > 1 were selected for further functional enrichments. To perform pathway enrichment associated with DEGs, we used the gene set enrichment analysis (GSEA) method based on HALLMARK gene sets from MSigDB database with the clusterProfiler package of R. The bioinformatic analyses were conducted based on R version 3.6.3.

### Statistics

Statistical analysis was performed with GraphPad Prism software. Data were presented as mean ± SEM of three independent experiments. Differences between groups were analyzed using Student's t-test or one-way analysis of variance (ANOVA), followed by post hoc tests for multiple comparisons when applicable. Statistical significance was set at p < 0.05.

## Results

### Upregulation of inflammasomes components in HT patients

**Fig. 1:**
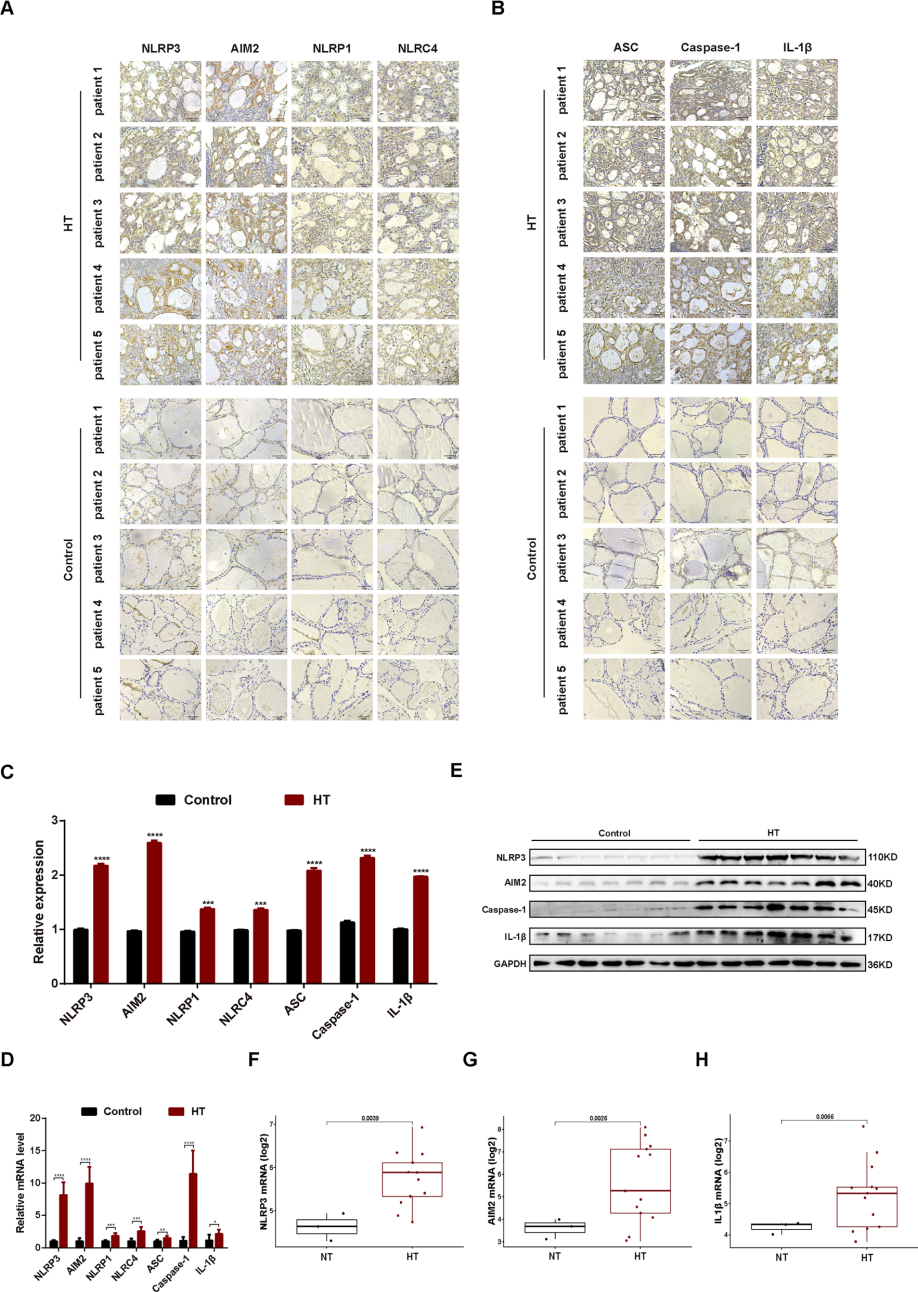
Upregulation of inflammasomes components in HT patients. **A** Immunohistochemical staining images of NLRP3, AIM2, NLRP1, and NLRC4 in thyroid tissue sections from patients with HT and control subjects. **B** Immunohistochemical staining images of ASC, caspase-1 and IL-1β in thyroid tissue sections from patients with HT and control subjects. **C** Quantified expression levels of NLRP3, AIM2, NLRP1, NLRC4, ASC, caspase-1, IL-1β in thyroid tissue sections from HT patients and controls using Image J (n = 5 for each group). Three views were randomly selected in each subject. *** *p* < 0.001, ****p < 0.0001, versus control. **D** The mRNA expression levels of NLRP3, AIM2, NLRP1, NLRC4, ASC, caspase-1 and IL1β in thyroid tissues from HT patients and controls (n = 7 for each group). *p < 0.05, **p < 0.001, ***p < 0.001, ****p < 0.0001, versus control. The relative mRNA expression levels were corrected to those of controls. **E** Western blot analysis of NLRP3, AIM2, Caspase-1, IL-1β in thyroid tissue from HT patients and controls (7 specimens for each group). **F**, **G**, **H** Comparison of NLRP3, AIM2, and IL1-β expressions between HT and normal thyroid (NT) tissues in GSE138198 dataset

To assess inflammasome dysregulation in HT patients, we conducted H&E staining and immunohistochemistry (IHC) assays on five HT tissues and five normal thyroid tissues. Pathological alterations in HT patient thyroid tissue revealed thyroid follicle destruction, extensive lymphocyte infiltration, and the formation of lymphoid follicles (Fig. S1). As displayed in Fig. 1A, C, expression of inflammasome components such as NLRP3, AIM2, NLRC4, and NLRP1 were elevated in thyroid follicular cells (TFCs) of HT patients compared to normal tissues. Interestingly, markers indicative of inflammasome activation-associated pyroptosis, including ASC, Caspase-1, and IL-1β, also demonstrated an increase (Fig. 1B, C). In terms of transcription, RT-qPCR yielded comparable results, showing an upsurge in mRNA for all examined inflammasome components and pyroptosis effectors in HT patients (Fig. 1D). The marked increase of NLRP3, AIM2, Caspase-1, and IL-1β found in prior tests was further corroborated by western blot analysis of fresh tissues from the same patients (Fig. 1E). To validate our results, we also examined a public transcriptome dataset, GSE138198, containing 3 normal thyroid tissues and 13 HT tissues; the expression profile of NLRP3, AIM2, and IL-1β also indicated aberrant inflammasome activation and pyroptosis in HT patients (Fig. 1F–H). Our data points to abnormal activation of various inflammasome components in HT patients, with the most significantly altered known pyroptosis mediators being NLRP3 and AIM2, suggesting the potential role of inflammasome-linked pyroptosis in HT pathogenesis.

### Simultaneous screening of upregulated USPs and activated inflammasome components in TFC cell line upon stimulation with inflammatory cytokines

**Fig. 2:**
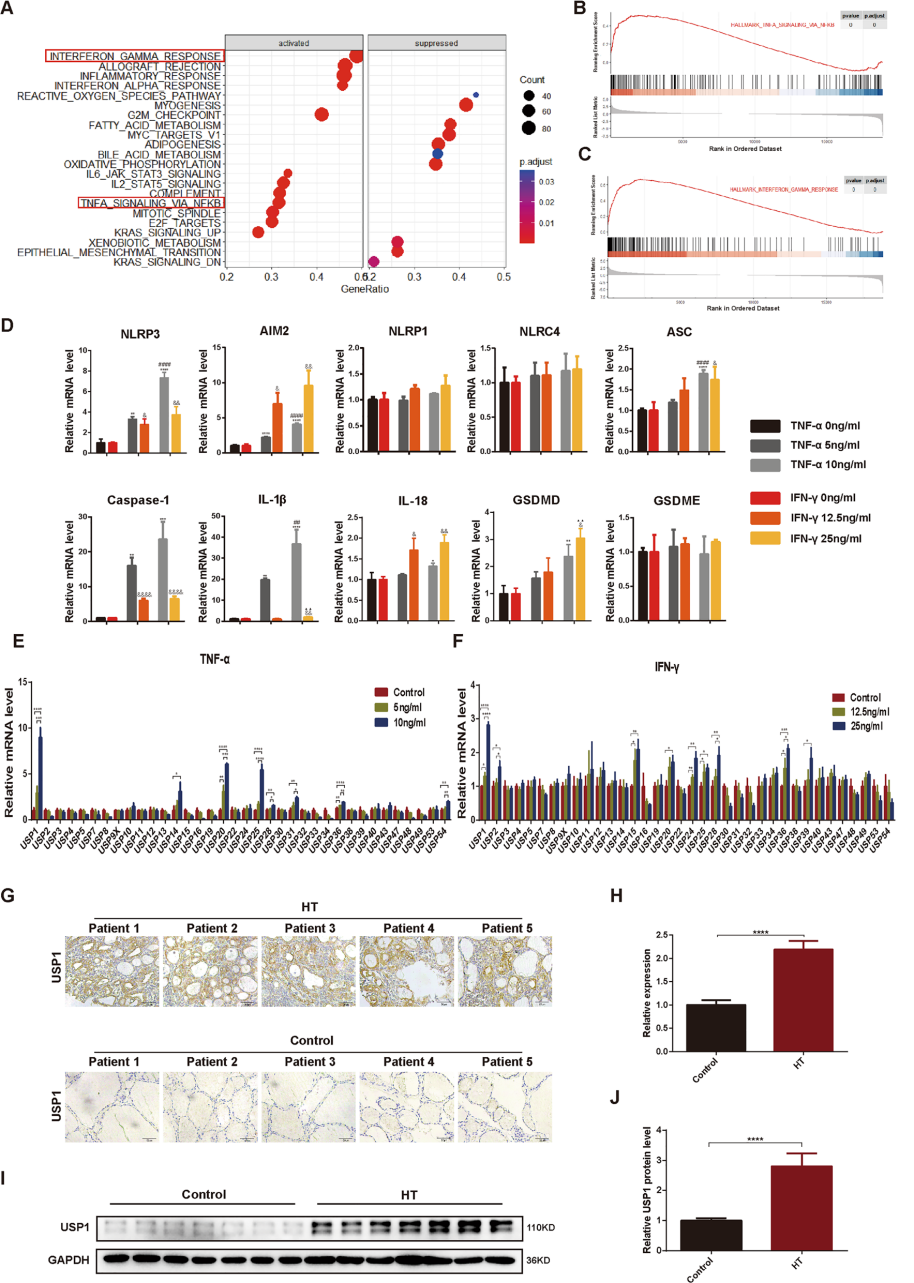
Simultaneous screening of upregulated USPs and activated inflammasome components in TFC cell line upon stimulation with inflammatory cytokines. **A** GSEA showing hallmark gene sets of DEGs in HT versus normal thyroid tissues in GSE138198 dataset. **B** GSEA of TNF-α response pathway. **C** GSEA of IFN-γ response pathway. **D** The relative mRNA levels of NLRP3, AIM2, NLRP1, NLRC4, ASC, caspase-1, IL-1β, IL-18, GSDMD and GSDME in Nthy-roi 3-1 cells upon stimulation with TNF-α or IFN-γ. *p < 0.05, **p < 0.01, ***p < 0.001, ****p < 0.0001, versus TNF-α 0 ng/ml; ^##^*p* < 0.01, ^####^*p* < 0.0001, versus TNF-α 5 ng/ml; ^&^*p* < 0.05, ^&&^*p* < 0.01, ^&&&&^*p* < 0.0001, versus IFN-γ 0 ng/ml; ^▲▲^*p* < 0.01, ^▲▲▲▲^*p* < 0.0001, versus IFN-γ 12.5 ng/ml. Data were obtained from three independent experiments. **E**, **F** Real-time PCR assessment of mRNA expression of USPs in Nthy-roi 3-1 cells after TNF-α or IFN-γ stimulation. *p < 0.05, **p < 0.01, ***p < 0.001, ****p < 0.0001. Data were obtained from three independent experiments. **G** Immunohistochemical staining images of USP1 in thyroid tissue sections from HT patients and controls. **H** Quantified expression levels of USP1 in thyroid tissue sections from HT patients and controls using Image J (n = 5 for each group). Three views were randomly selected in each subject. **** *p* < 0.0001, versus control. **I** Western blot analysis of USP1 in thyroid tissue from HT patients and controls. **J** Quantified expression levels of USP1 in thyroid tissue from HT patients and controls using Image J (n = 7 for each group). ****p < 0.0001, versus control

Given that HT is an immune-associated condition, we further examined the dataset GSE138198 to ascertain the inflammatory signaling pathways predominantly implicated in HT progression. In alignment with our hypothesis, gene enrichment and GSEA results revealed disruptions in multiple inflammatory pathways including IFN-γ, IL6-JAK-STAT3, IL2-STAT5, and TNFα in HT patients. Notably, inflammasome activation necessitated pathways like IFN-γ and TNF-α (Fig. 2A–C). With IHC, we also detected heightened levels of IFN-γ (Fig. S2A) and TNF-α (Fig. S2B) in thyroid tissues from HT patients. To delve into the role of IFN-γ and TNF-α in inflammasome-related pyroptosis within TFCs, we stimulated the Nthy-roi 3-1 cell line with varying doses of IFN-γ and TNF-α. Both cytokines led to a dose-dependent upregulation of mRNA levels for NLRP3 and AIM2, while other inflammasome components like NLRP1 and NLRC4 remained unaffected. Similarly, there was an increase in the expression of pyroptosis effectors ASC, Caspase-1, IL-1β, IL-18, and GSDMD, but GSDME remained unaffected (Fig. 2D). These observations suggest that aberrant activation of inflammatory pathways could be a deleterious factor provoking inflammasomes and pyroptosis in TFCs during HT development. Further scrutiny spotlighted the upregulated USPs during TNF-α stimulation alongside inflammasome induction. RT-qPCR screening in Nthy-roi 3-1 cells unveiled increased expression of several USPs, including USP1, USP14, USP20, USP25, USP28, USP31, USP36, and USP54, with USP1 exhibiting the most significant change (Fig. 2E). A marked surge was also observed in USP1 expression upon gradient IFN-γ stimulation (Fig. 2F). Both IHC and western blot assays confirmed a higher USP1 expression in HT patients with severe thyroid follicle destruction and lymphocyte infiltration (Fig. 2G–J), suggesting that USP1 could potentially contribute to inflammasome and pyroptosis initiation in the pathogenesis of HT.

### USP1 facilitates cytokines induced inflammasome activation and pyroptosis in TFCs

**Fig. 3:**
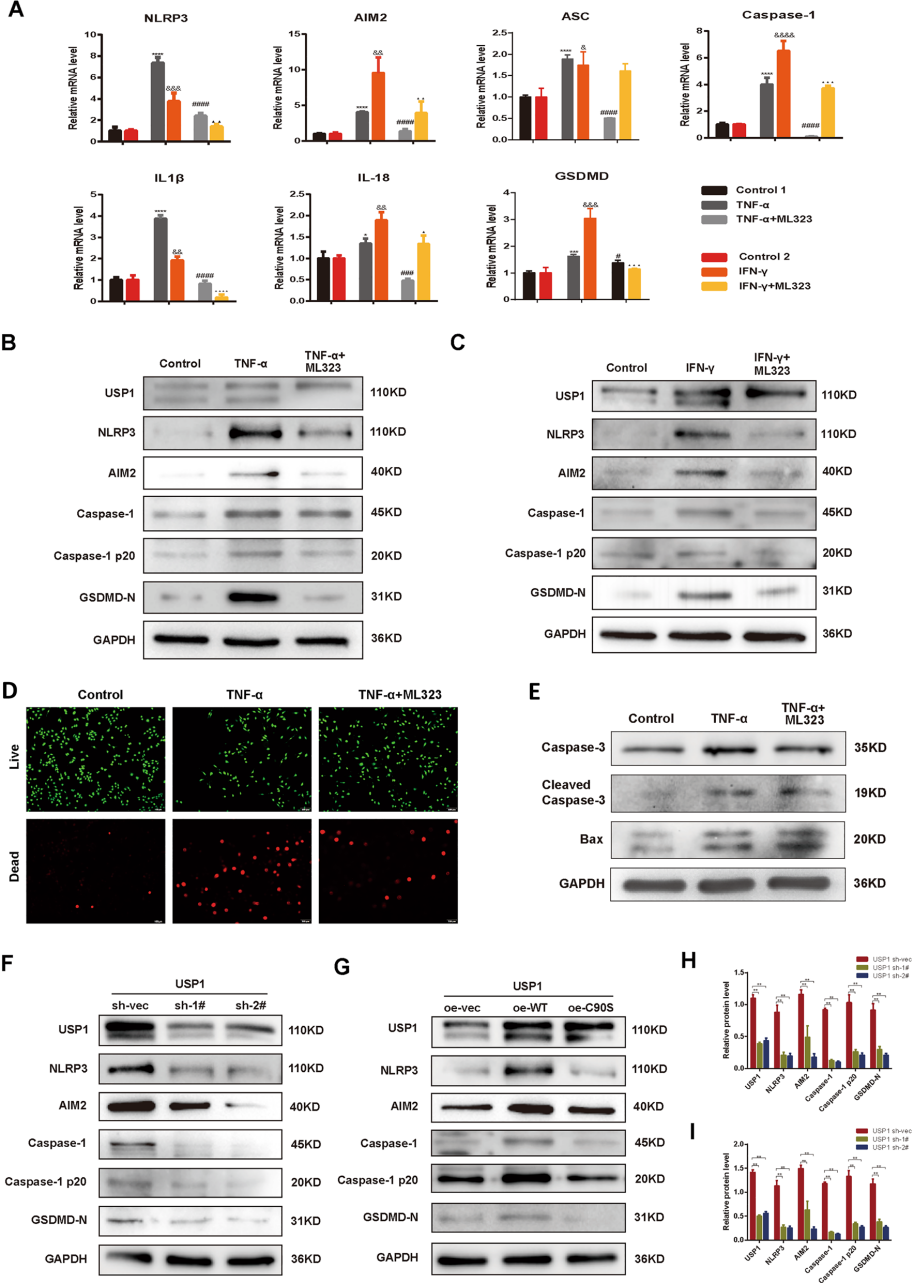
USP1 facilitates cytokines-induced inflammasome activation and pyroptosis in TFCs. **A** Real-time PCR measurement of mRNA levels of NLRP3, AIM2, ASC, caspase-1, IL-1β, IL-18, and GSDMD following different stimulations. TNF-α, 10 ng/ml; IFN-γ, 25 ng/ml; ML323, 50 μm. *p < 0.05, ***p < 0.001, ****p < 0.0001, versus control 1; ^#^*p* < 0.05, ^###^*p* < 0.001, ^####^*p* < 0.0001, versus TNF-α; ^&^*p* < 0.05, ^&&^*p* < 0.01, ^&&&^*p* < 0.001, ^&&&&^*p* < 0.0001, versus control 2; ^▲^*p* < 0.05, ^▲▲^*p* < 0.01, ^▲▲▲^*p* < 0.001, ^▲▲▲▲^*p* < 0.0001, versus IFN-γ. Data were obtained from three independent experiments. **B**, **C** Western blotting of NLRP3, AIM2, caspase-1, caspase-1 p20 and GSDMD-N in the indicated groups. TNF-α, 10 ng/ml; IFN-γ, 25 ng/ml; ML323, 50 μm. **D** Calcein AM/EthD-1 double staining of Nthy-roi 3-1 cells under different conditions. TNF-α, 10 ng/ml; ML323, 50 μm. **E** Western blotting of caspase-3, cleaved caspase-3 and Bax in the indicated groups. TNF-α, 10 ng/ml; ML323, 50 μm. **F** Protein levels of NLRP3, AIM2, caspase-1, caspase-1 p20, and GSDMD-N in normal, USP1-deficient Nthy-roi 3-1 cells assessed by Western blotting. **G** Protein level of NLRP3, AIM2, caspase-1, caspase-1 p20 and GSDMD-N in Nthy-roi 3-1 cells transfected with USP1 overexpression plasmid and USP1 C90S mutant plasmid. **H, I** Quantitative analysis of the bands conducted using ImageJ. *p < 0.05, **p < 0.01. Data were obtained from three independent experiments

Observing the concurrent upregulation of USP1 and inflammasome-associated pyroptosis in TFCs, the query arises whether the augmented USP1 serves as a driving mechanism or is merely a concomitant process. To unravel this uncertainty, we exposed Nthy-roi 3-1 cells to TNF-α or IFN-γ, along with the USP1 inhibitor ML323. Our investigations revealed that ML323 mitigated the cytokine-induced elevations in NLRP3, AIM2, ASC, caspase-1, caspase-1 p20 (activated form), GSDMD, IL-1β, and IL-18 at either mRNA or protein levels (Fig. 3A–C). Additionally, ML323 also hindered TNF-α-triggered cell death, primarily due to the inhibition of pyroptosis, as apoptosis, the primary mode of cell death, remained unaffected (Fig. 3D, E). Assays involving knockdown and overexpression of USP1 and its enzymatically deficient mutant (C90S) in Nthy-roi 3–1 cells further reinforced our findings that functional USP1 modulates the expression of inflammasomes and pyroptosis (Fig. 3F–I). These results underscore the pivotal role of USP1 in instigating inflammasome activation and subsequent pyroptotic events in TFCs.

### USP1 enforces pyroptosis in TFCs by dual regulation of NLRP3 and AIM2

**Fig. 4:**
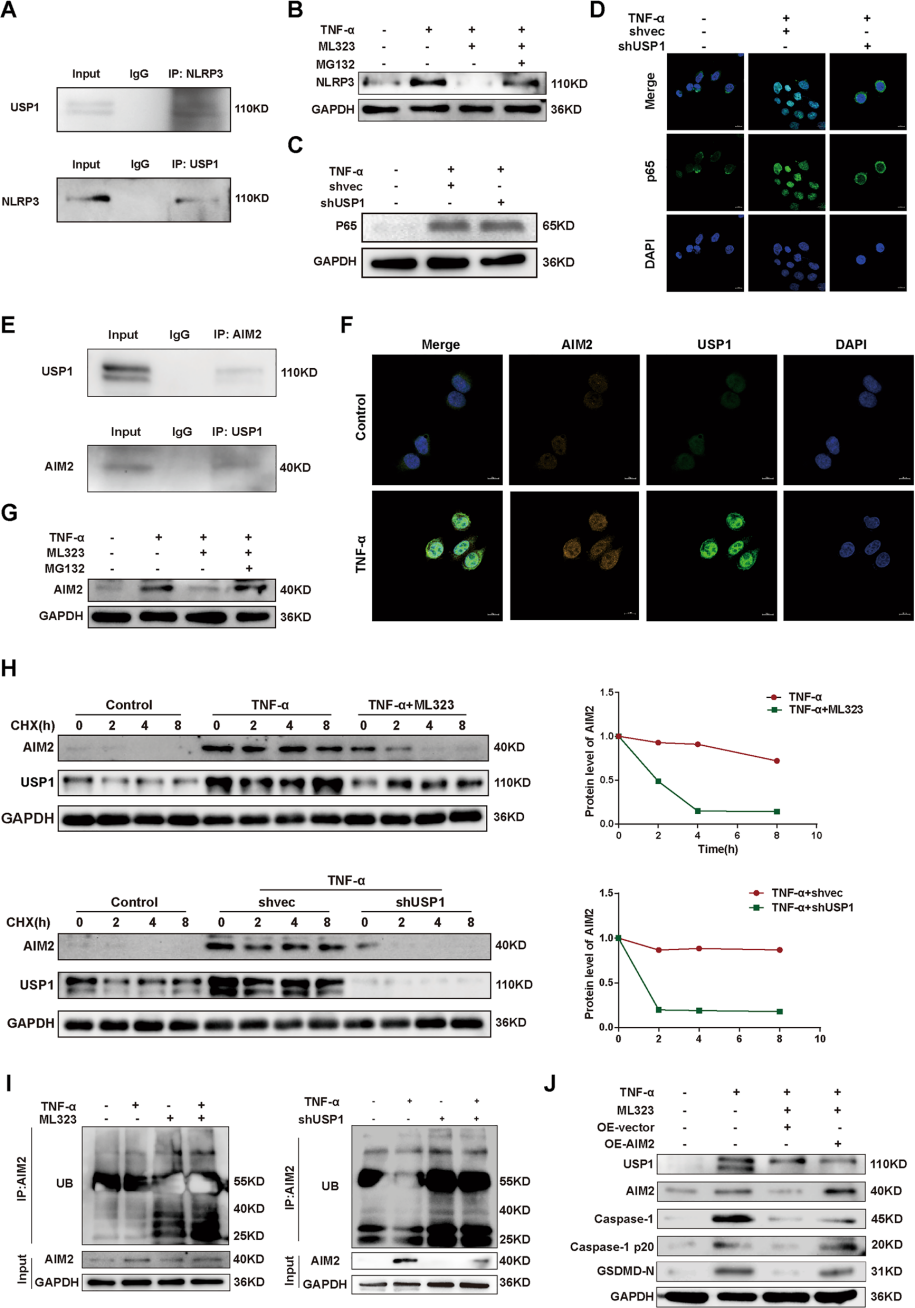
USP1 enforces pyroptosis in TFCs by dual regulation of NLRP3 and AIM2. **A** Co-immunoprecipitation (Co-IP) assay showing the endogenous interaction between USP1 and NLRP3 in Nthy-roi 3-1 cells. **B** Partial rescue of repressed NLRP3 expression by ML323 (50 μM) through treatment with the proteasome inhibitor MG132 (10 μM). **C** Protein expression of p65 upon TNF-α and USP1 knockdown. **D** Immunofluorescence analysis of p65 upon TNF-α and USP1 knockdown. **E** Co-IP assay revealing the endogenous interaction between USP1 and AIM2 in Nthy-roi 3-1 cells. **F** Immunofluorescence analysis demonstrating colocalization of USP1 with AIM2. **G** Partial rescue of repressed AIM2 expression by ML323 (50 μM) through treatment with the proteasome inhibitor MG132 (10 μM). **H** Determination of AIM2 protein half-life in Nthy-roi 3-1 cells under different treatments using cycloheximide (CHX, 100 μg/ml) at indicated time intervals. **I** Increased ubiquitination level of AIM2 upon inhibition of USP1 in Nthy-roi 3-1 cells. **J** Enhanced protein levels of caspase-1 p20 and GSDMD-N through overexpression of AIM2 in TNF-α + ML323 treated Nthy-roi 3-1 cells. TNF-α, 10 ng/ml; ML323, 50 μM

To further investigate the regulatory role of USP1 in inflammasome activation and pyroptosis in TFCs, we performed endogenous Co-immunoprecipitation (CoIP) assays to examine the interaction between USP1 and the key components of inflammasomes (NLRP3 and AIM2). The hypothesis was that USP1 could stabilize these components through its enzymatic function. Prior research in human macrophages has postulated that USP1 manipulates NLRP3 at both transcriptional and post-translational levels, encompassing direct deubiquitination of NLRP3 protein and regulation of the NLRP3-associated transcription factor p65 (Song et al. 2020). However, this study revealed that while an interaction between USP1 and p65 was detected, it was the USP1 binding cofactor UAF1, not USP1 itself, that played an essential role in stabilizing p65 protein (Song et al. 2020). Corroborating previous reports, we also evidenced the association between USP1 and NLRP3 in TFCs (Fig. 4A). Alongside, we noted that ML323-induced degradation of NLRP3 could be counteracted by the protease inhibitor MG132 (Fig. 4B), indicating the existence of USP1's deubiquitinating functionality on NLRP3 in TFCs. Interestingly, although p65 expression remained unaltered upon USP1 knockdown (Fig. 4C), TNF-α-induced nuclear accumulation of p65 markedly diminished (Fig. 4D), signifying USP1's exclusive role in governing NLRP3 transcription. The interaction between USP1 and AIM2 was confirmed via CoIP assay and immunofluorescence (Fig. 4E, F). Impeding USP1 notably curtailed TNF-α-propelled AIM2 expression, which could be restored by the addition of MG132 (Fig. 4G). Utilizing CHX to restrain de novo protein synthesis, we verified the attenuated protein stabilization of AIM2 associated with USP1 deficiency (Fig. 4H), which corresponded with amplified ubiquitination of AIM2 (Fig. 4I). Lastly, we confirmed TNF-α-induced pyroptosis in TFCs, as suggested by increased expression of caspase-1 p20 and GSDMD-N. ML323 treatment suppressed this effect, which was partially counteracted by enforced expression of AIM2 (Fig. 4J), underscoring the functional significance of the USP1-AIM2 axis in modulating pyroptosis in TFCs.

### USP1 driven pyroptosis is associated with HT pathogenesis

**Fig. 5:**
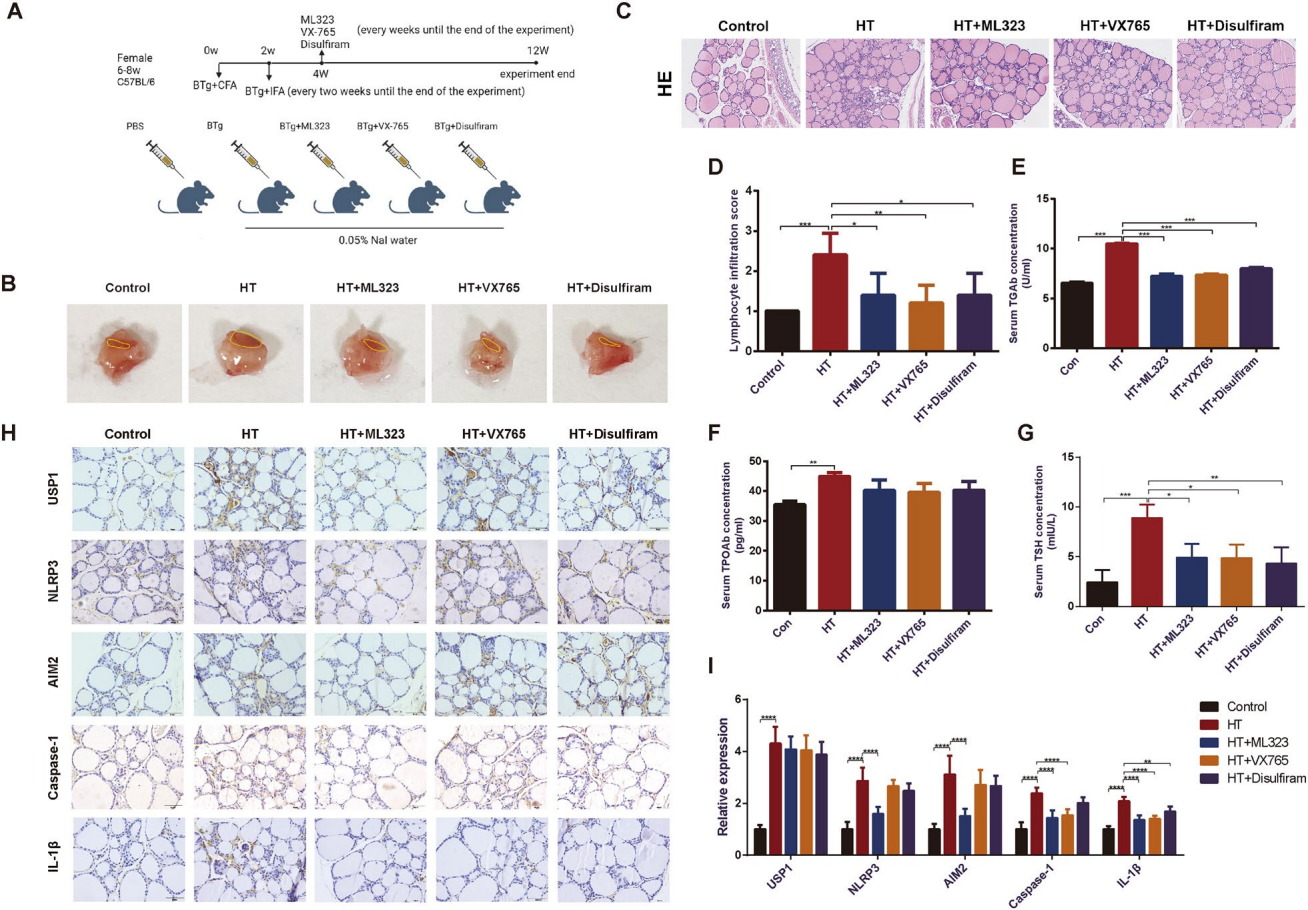
USP1 driven pyroptosis is associated with HT pathogenesis. **A** Experimental process of the animal model. BTg: bovine thyroglobulin. CFA: complete Freund’s adjuvant. IFA: incomplete Freund's adjuvant. **B**: Thyroid tissues (marked with yellow line) from mice. **C** H&E staining of mouse thyroid tissues. **D** Lymphocyte infiltration scores of mouse thyroid tissues. n = 6. **E**, **F**, **G** Expression of TGAb, TPOAb and TSH in plasma of animal models. *p < 0.05, **p < 0.01, ***p < 0.001. n = 6. Data were obtained from three independent experiments. **H** Immunohistochemical staining images of USP1, NLRP3, AIM2, caspase-1, and IL-1β in sections of mouse thyroid tissues. **I** Quantified expression levels of USP1, NLRP3, AIM2, caspase-1and IL-1β in thyroid tissue sections from mouse models using Image J. ** *p* < 0.01, ****p < 0.0001. n = 6

To assess the potential influence of USP1 on HT progression, a thyroglobulin-induced mouse model was established, following our prior demonstration of the correlation between USP1 and inflammasome-related pyroptosis. As illustrated in Fig. 5A, administering thyroglobulin and adjuvant for 12 weeks successfully induced HT in mice, as evidenced by an expanded thyroid volume, increased lymphocyte infiltration, and elevated serum TGAb, TPOAb and TSH levels (Fig. 5B–G). Utilizing IHC, we detected heightened levels of IFN-γ and TNF-α in thyroid tissues gathered from murine HT models (Fig. S3). Additionally, an upregulation of USP1, along with inflammasome components or pyroptosis effectors, was noted in HT mice (Fig. 5H). To scrutinize the role of TFCs' pyroptosis in HT development, pyroptosis inhibitors VX-765 (Caspase-1 inhibitor) (Sun et al. 2020) and disulfiram (GSDMD inhibitor) (Hu et al. 2020) were employed. Administering these inhibitors for eight weeks exhibited beneficial effects through reduction of thyroid volume, lymphocyte infiltration, serum TGAb, TPOAb and TSH levels, and attenuation of Caspase-1, IL-1β expression without impacting the expression of USP1 or inflammasomes upstream of pyroptosis (Fig. 5B–I), signifying the importance of TFCs' pyroptosis in HT development. To further comprehend the function of USP1 concerning pyroptosis-triggered HT pathogenesis, ML323 was injected weekly during the final 8 weeks. Data revealed that ML323 could significantly mitigate HT progression (Fig. 5B–G), akin to the pyroptosis inhibitors previously utilized. Furthermore, it suppressed the expression of inflammasomes (Fig. 5H, I). This indicates that the functional role of USP1 in HT development is largely moderated by inflammasome activation and pyroptosis.

## Discussion

HT is a persistent ailment with a notable prevalence among women of reproductive age (Mincer et al. 2022). The presence of hypothyroidism as a comorbidity in HT patients can significantly increase the risk of developing cardiovascular disease, and could potentially disrupt the neurological development of their progeny (Chen et al. 2015; Lee and Pearce 2022). Therefore, there is a critical need to explore novel therapeutic modalities that can effectively impede the advancement of this condition.

Recent studies suggest that inflammasomes mediated pyroptosis contributes to HT progression, with upregulated NLRP1, NlRP3, NLRC4, and AIM2 inflammasome components observed in HT patients (Guo et al. 2018). Our research corroborated these findings and went further to identify NLRP3 and AIM2 as the most significantly altered inflammasomes during HT development. By analyzing gene expression datasets from public sources, we found that two pro-inflammatory signalings, TNF-α and IFN-γ, which related to inflammasome activation and pyroptosis (Guo et al. 2018; Ezquerro et al. 2019; Yang et al. 2023), were highly enriched in HT tissues. Subsequent experiments confirmed that TNF-α and IFN-γ could induce inflammasome activation and pyroptosis in TFCs. As these cytokines are known to cause oxidative DNA damage and DNA strand breaks in multiple cell types during the pathogenesis of various immune disorders (Yang et al. 2019; Collins et al. 2020; Pereira-Lopes et al. 2015), we propose that aberrant cytoplasmic DAMPs (especially dsDNA) triggered by TNF-α or IFN-γ may initiate TFCs' pyroptosis during HT progression and be sensed by NLRP3 or AIM2 directly or indirectly.

Intracellular proteins undergo various post-translational modifications that result in dynamic changes in their status (Popovic et al. 2014; Wang et al. 2022). Post-translational deubiquitination is a well-known process associated with several autoimmune diseases (Parihar and Bhatt 2021). For example, USP4 has been shown to interact with and deubiquitinate RORγt, promoting its function and IL-17A transcription in rheumatic heart disease (Yang et al. 2015); similarly, USP7 has been found to promote lupus nephritis by regulating NF-κB p65 signaling via JMJD3 stabilization (Zhang et al. 2021). However, the role of deubiquitination or deubiquitinating enzymes in HT development is not well established. In this study, we screened the USP family of deubiquitinating enzymes in TFCs following stimulation with IFN-γ or TNF-α and found that USP1 was the most abundantly upregulated member. We also observed abnormal expression of USP1 in TFCs of HT patients. In vivo assays confirmed that inhibition of USP1 significantly improved HT progression, similar to the effects observed following pyroptosis suppression. These findings suggest that USP1 may contribute to HT progression by stimulating inflammasome and pyroptosis in TFCs.

**Fig. 6:**
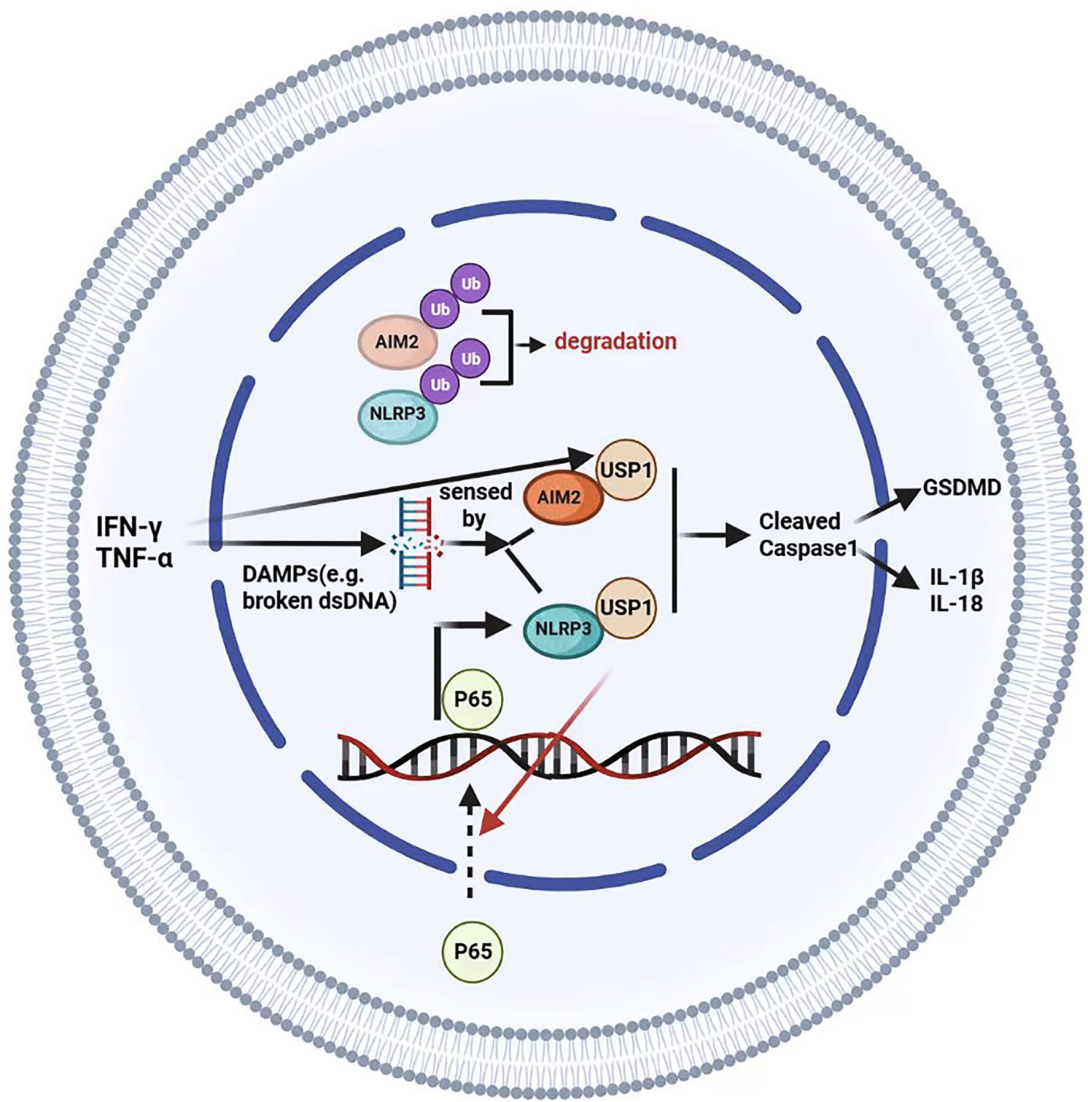
The schematic diagram of the study. USP1 promotes inflammasome-related pyroptosis in TFCs by stabilizing NLRP3/AIM2 and transcriptional activation of NLRP3 through facilitating P65 nuclear accumulation

Previous studies have demonstrated inappropriate NLRP3 inflammasome activation resulting from the action of USPs (Palazon-Riquelme et al. 2018; Liu et al. 2021). Specifically, in human macrophages, the UAF1/USP1 complex binds and stabilizes NLRP3 through deubiquitination, while UAF1 regulates NLRP3 transcription through the stabilization of NF-κb, independent of USP1 function (Song et al. 2020). In this study, we identified the interaction between USP1 and NLRP3 in TFCs and confirmed that USP1 exclusively promotes NLRP3 transcription by enhancing the nuclear transportation of p65. Similarly, we found that USP1 could interact with AIM2 and deubiquitinate it, promoting pyroptosis in TFCs. Inhibition of USP1 led to the degradation of AIM2 protein and a subsequent decrease in pyroptosis in TFCs; this effect was partially rescued by AIM2 overexpression. Interestingly, we also observed elevated mRNA levels of AIM2 triggered by IFN-γ or TNF-α, which were reversed by USP1 inhibition. These findings suggest that USP1 regulates AIM2 and NLRP3 at both transcriptional and post-translational levels, facilitating pyroptosis in TFCs and HT progression (Fig. 6).

In concluding, our investigation underscores a regulatory role of USP1 in inflammasome activation and pyroptosis in thyrocytes under HT pathogenesis. This elucidates further on HT and proposes that USP1 inhibition could be an innovative therapeutic approach for HT management. However, there are limitations to the present study. Firstly, we employed female human samples and animal models for experimental consistency due to the higher prevalence of females with the condition. The discrepancy in morbidity between sexes might be linked to an imbalance of sex hormones, which potentially impacts immune system functionality. For instance, testosterone and the precursors of androgens have been found to protect against thyroid autoimmunity (Krysiak et al. 2019, 2021). Therefore, it remains uncertain whether a similar mechanism wherein USP1 accelerates pyroptosis exists in males, and if there is any gender hormone regulatory potential in USP1—these require further determination. Additionally, the C57BL/6 mice model was selected based on referenced literature (Jia et al. 2021; Choi et al. 2014; Lin et al. 2019). We did not use more classical HT mice models such as CBA/J or NOD.H2h4 mainly because of laboratory constraints and extended delivery times. However, even the most widely used HT mice model, NOD.H2h4, fails to fully replicate the distinct human morbidities between sexes (Rasooly et al. 1996), underscoring the limitations of a sole model. Consequently, future work will include the validation of our results across various strains of mice and rats.

### Supplementary Information

Supplementary Material 1.

## Data Availability

The datasets used and/or analyzed during the current study are available from the corresponding author upon reasonable request.
