# Supplementary material for: Multi-regulatory potency of USP1 on inflammasome components promotes pyroptosis in thyroid follicular cells and contributes to the progression of Hashimoto's thyroiditis
Source: Mol Med. 2024 Aug 12;30:121. doi: 10.1186/s10020-024-00885-w (PMC11318162; doi:10.1186/s10020-024-00885-w)
Supplement: Supplementary file 1 — Supplementary Material 1. [file 10020_2024_885_MOESM1_ESM.pdf]

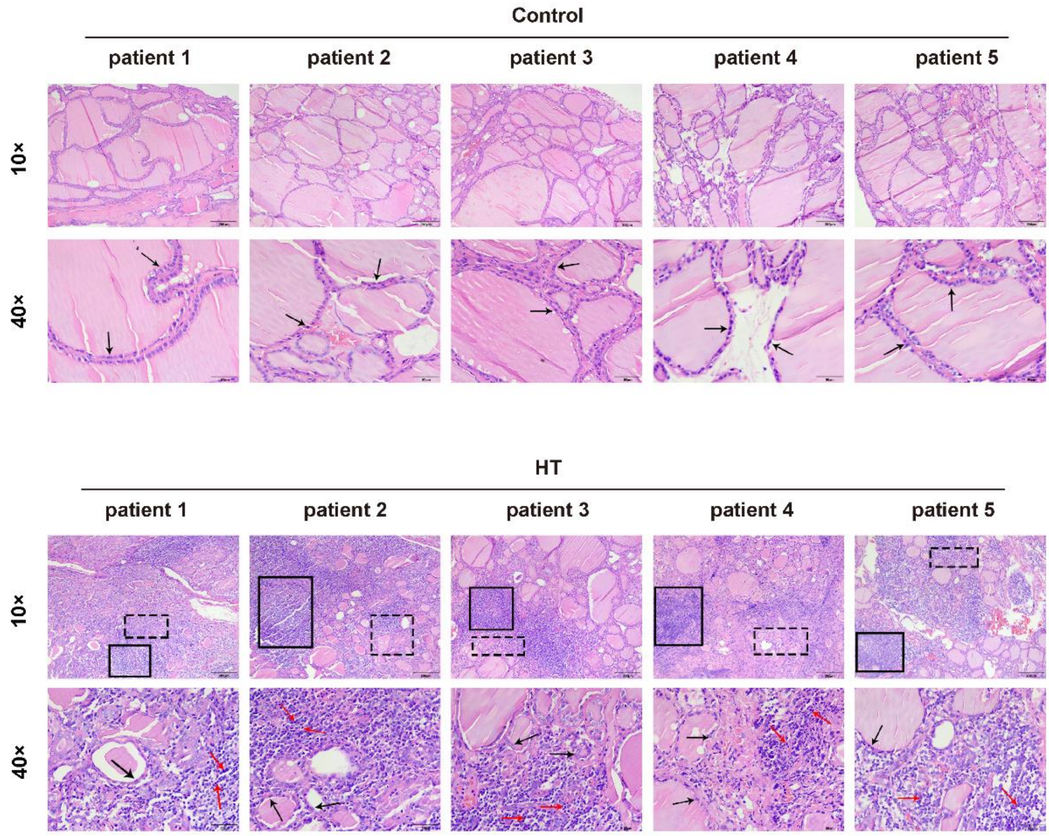

Figure S1. Pathological characteristics of thyroid tissues derived from HT patients and control subjects.

H&E staining was performed on thyroid tissue sections obtained from HT patients and control subjects. Solid line boxes illustrate the formation of lymphoid follicles, while dotted line boxes highlight instances of thyroid follicle destruction. Black arrows mark lymphocytes, identifiable by their high nuclear-to-cytoplasmic ratio and darkly stained nuclei. Red arrows indicate thyroid follicular cells (TFCs).

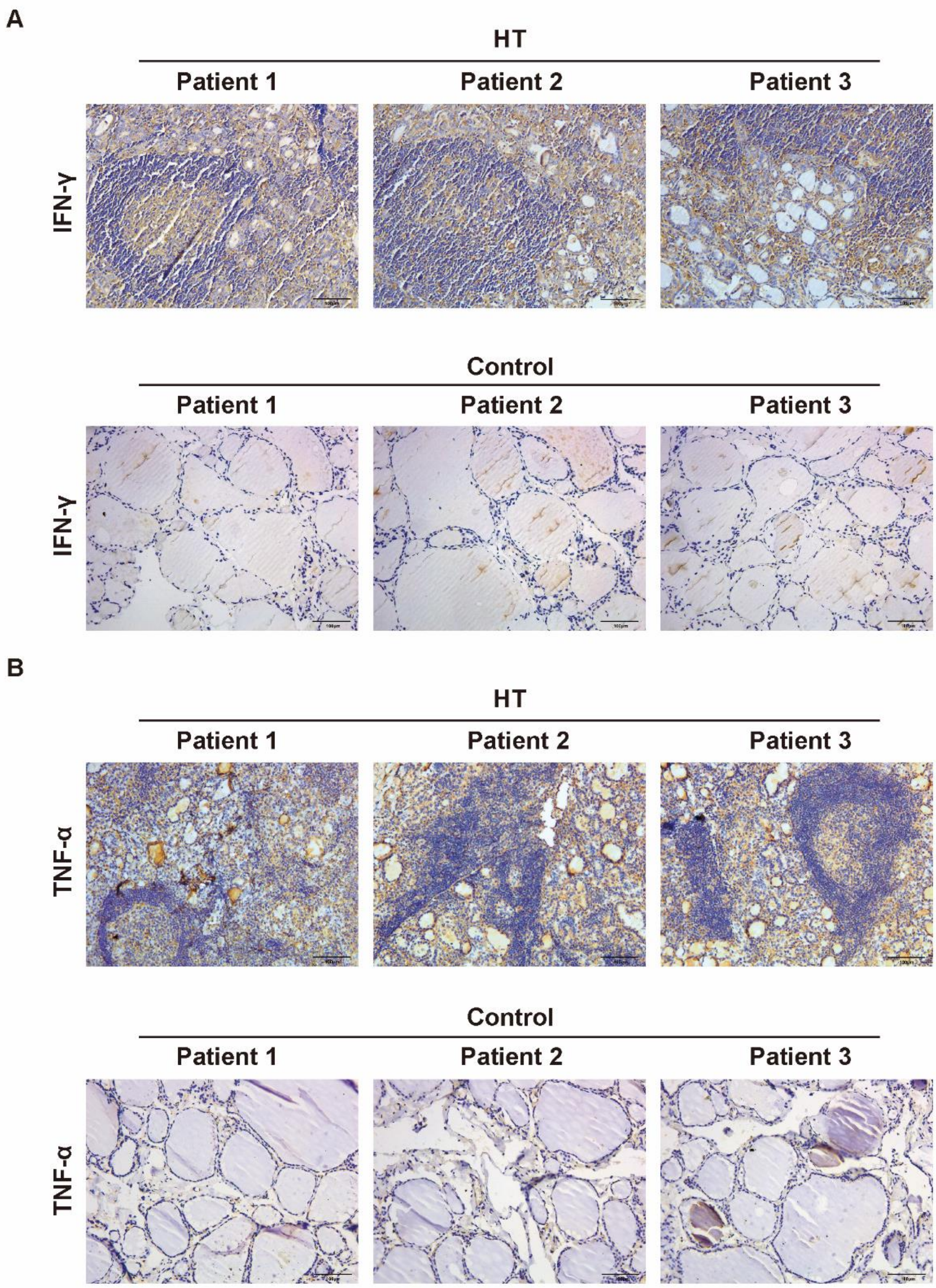

Figure S2. Elevated IFN-γ and TNF-α in thyroid tissues from patients with HT. Immunohistochemical staining images of IFN-γ (A) and TNF-α (B) in thyroid tissue sections from HT patients and controls.

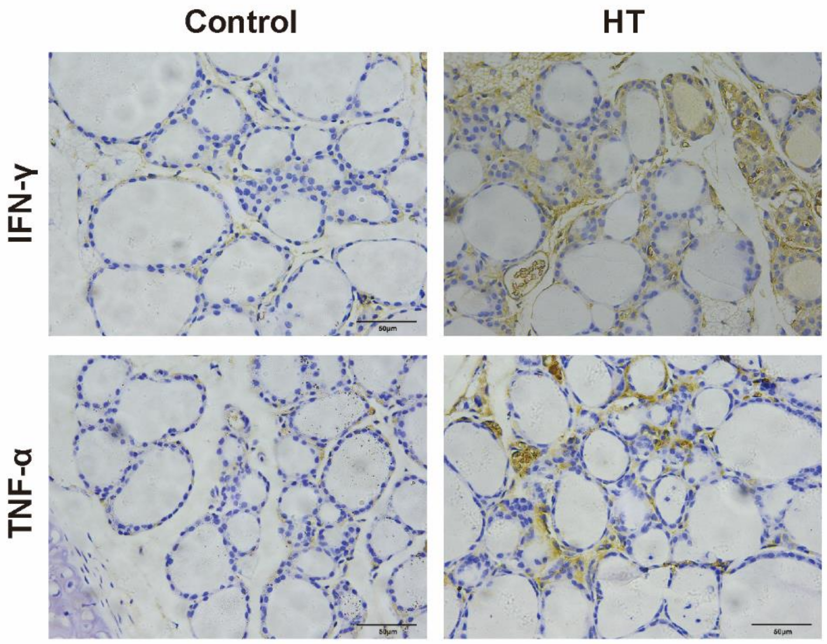

Figure S3. Elevated IFN-γ and TNF-α in thyroid tissues from mouse models with HT. Immunohistochemical staining images of IFN-γ and TNF-α in sections of mouse thyroid tissues.

Table S1. Primer sequences for real-time PCR.

Gene	Sequence(5'-3')
NLRP3	F: AGGGATGAGAGTGTTGTGTGAAACG
	R: GCTTCTGGTTGCTGCTGAGGAC
AIM2	F: CTACAGTGGCTACAGAAAAGGA
	R: CAGATTCAGCATCTAACACACG
NLRP1	F: GGCTACTGAGGTAGTTGACAAA
	R: GTCCCACACACAGAATTCAATC
NLRC4	F: TCCCACAAGTTTGATTTCGAAC
	R: GGCTCATGAGACGTCAATAAAC
ASC	F: CTCAAGAAGTTCAAGCTGAAGC
	R: TAGGTCTCCAGGTAGAAGCTG
Caspase-1	F: GAAGAAACACTCTGAGCAAGTC
	R: GATGATGATCACCTTCGGTTTG
IL-1β	F: CCAGGGACAGGATATGGAGCA
	R: TTCAACACGCAGGACAGGTACAG
IL-18	F: CTGCCACCTGCTGCAGTCTA
	R: TCTACTGGTTCAGCAGCCATCTTTA
GSDMD	F: TTGAAGAACTGAGTGTGGACAGAGC
	R: TGGTGGTGTGTGCGTTGGAATG
GSDME	F: TTTGCATTCATAGACATGCCAG
	R: GTTCATCATCAAATAGGACCGC

F, forward; R, reverse

Table S2. Key Resource

REAGENT or RESOURCE	SOURCE	IDENTIFIER
Antibodies		
Rabbit Polyclonal anti-NLRP3	Wanleibio	Cat# WL02635, RRID:AB_2935859
Rabbit Polyclonal anti-NLRP1	ABclonal	Cat# A16212, RRID:AB_2763666
Rabbit Polyclonal anti-NLRC4	ABclonal	Cat# A7382, RRID:AB_2767914
Rabbit Polyclonal anti-TNF-α	ABclonal	Cat# A11534, RRID:AB_2758597
Rabbit Polyclonal anti-Caspase-1	ABclonal	Cat# A0964, RRID:AB_2757485
FITC Goat Anti-Rabbit IgG (H+L)	ABclonal	Cat# AS011, RRID:AB_2769476
Cy3 Goat Anti-Mouse IgG (H+L)	ABclonal	Cat# AS008, RRID:AB_2769088
Mouse Monoclonal anti-AIM2	Santa Cruz	Cat# sc-515514
Mouse Monoclonal anti-IL-1β	Santa Cruz	Cat# sc-32294 , RRID:AB_627790
Mouse Monoclonal anti-ASC	Santa Cruz	Cat# sc-514414, RRID:AB_2737351
Mouse Monoclonal anti-UB	Santa Cruz	Cat# sc-271289, RRID:AB_10611436

Mouse Monoclonal anti-BAX	Santa Cruz	Cat# sc-7480, RRID:AB_626729
Rabbit Polyclonal anti-USP1	Proteintech	Cat# 14346-1-AP, RRID:AB_2214314
Rabbit Polyclonal anti-IFN-γ	Proteintech	Cat# 15365-1-AP, RRID:AB_2123037
Rabbit Polyclonal anti-GAPDH	Proteintech	Cat# 60004-1-Ig, RRID:AB_2107436
Rabbit Monoclonal anti-GSDMD-N	Abcam	Cat# ab215203, RRID:AB_2916166
Rabbit Monoclonal anti-p65	CST	Cat# 3033 (also 3033S, 3033L, 3033P), RRID:AB_331284
Rabbit Polyclonal anti-Caspase-3	CST	Cat# 9662, RRID:AB_331439
Rabbit Polyclonal anti- Cleaved Caspase-3	CST	Cat# 9661 (also NYUIHC-314, 9661S, 9661L), RRID:AB_2341188
Goat anti-rabbit IgG-HRP	Southernbiotech	Cat# 4030-05, RRID:AB_2687483
Rabbit anti-mouse IgG-HRP	Southernbiotech	Cat# 6170-05, RRID:AB_2796243
Chemicals, peptides, and recombinant proteins		
CHX	Sigma	66-81-9
Complete Freund's adjuvant	Sigma	F5581
Incomplete Freund's adjuvant	Sigma	F5506
bovine thyroglobulin	Bioss	Bs-0291P
ML323	MCE	HY-17543
VX765	MCE	HY-13205
Disulfiram	MCE	HY-B0240
MG132	MCE	HY-13259
TNF-α	R&D	10291-TA-020
IFN-γ	R&D	10067-IF-025
Deposited data		
GSE138198	National Institutes of Health (NIH)	https://www.ncbi.nlm.nih.gov/geo/query/acc.cgi?acc=GSE138198
Experimental models: Organisms/strains		
Eight-weeks-old female C57BL/6 mice	Laboratory Animal Center of Nantong University	
Oligonucleotides		
See Table S1 for the primers sequences	This paper	N/A
Software and algorithms		
ImageJ	National Institutes of Health (NIH)	https://imagej.net/ij/
GraphPad Software	San Diego, CA	https://www.graphpad.com
Other		
The LIVE/DEAD Viability/Cytotoxicity kit	Thermo	L3224

Mouse anti-thyroid-globulin antibody, TGAB ELISA Kit	CUSABIO	CSB-E09543m
Mouse Thyroid Stimulating Hormone (TSH) ELISA Kit	Jianglaibio	JL20301
